# Scutari—March, 1863

**Published:** 1863-10

**Authors:** J. N. Radcliffe


### Art. XI.—SCUTARI:—MARCH, 1863.

By J. N. Radcliffe.

I had an opportunity of visiting Scutari early in the spring of the present year. Twice before I had climbed the hill from the barrack-pier, and rambled through the vast building on its summit, and the wards of the general hospital—once in the spring of 1855, before the final traces of the past winter had been altogether effaced; once in the summer of the same year, when the then two great hospitals afforded succour, befitting a great nation, to the sick and wounded from the seat of war. Both visits were too brief to give me much familiarity with the hospitals and their vicinity, and my recent briefer pilgrimage lacks that interest which the memories evoked by a more thorough knowledge would have given to it. But Scutari is a sacred spot to the Englishman—sacred from the stupendous misery which it once overshadowed, and of which the sad remembrances have as yet been scarcely blunted by time—sacred from that quiet resting-place beneath which rests a hecatomb of England's bravest sons. It may be then that the notes of a passing visit will possess for some a momentary attraction.

There was so little change at the barrack-pier, with its fringe of caiques and caidjees—in the pathway up the ascent—in the tumble-down buildings at the bend of the road—in the stray Turkish soldier or two gossiping with a peasant over a chibouque—in the shaggy, wolf-like dogs slouching over the waste ground before the wide-extended front of the great barracks—in the general appearance of the barracks themselves, that it was difficult to get rid of a vague feeling of wonder that there were no red-jackets visible.

The same feeling had possessed me on the opposite shore. Accustomed to Constantinople in the height of the Russian war, many of the localities I had never seen without the concomitants of British or French men-of-war's men or soldiers. Turning well-known corners, it seemed natural to expect to stumble upon a diminutive French sentry standing in the shadow of his tall musket, or a knot of Zouaves airing their free-and-easy discipline for the benefit of the natives. And for the moment the delusion was complete. On first landing, I had scarcely struggled through the parti-coloured crowd at the foot of the bridge, and filling the new street leading to it at the Galata side, and begun to ascend the hill to Pera, when to my amazement I beheld lounging along before me three veritable Zouaves, perfect in costume from head to foot. There was the jaunty white turban with superabundant tassel, the dainty blue jacket, the voluminous red inexpressibles, the yellow gaiters. Amazed, I pushed hastily in advance, faced these strange anachronisms, and, lo! — unmitigated Turks. Truly Constantinople was changed and changing. It retains its miserable tenements, its sewer-like streets, its horrible filth, its wondrous beauties—for filth is not at all times inconsistent with beauty, and judiciously treated will often enrich the tone and colour of the canvas. Happily, even the transcendental pre-Raphaelite has not yet succeeded, when limning mud, in limning the odour also. Ten years ago, the chief indication of occidental infection in the Turk was the caricature of a Western uniform adopted for the army. But the present Sultan has displayed a juster appreciation of the examples which may be best copied from a more advanced civilization. He has clothed his troops, horse and foot, in their national costume—that is to say, *à la Zouave;* and he has built beneath the shadow of the great Mosque of Sultan Achmed an "Industrial Exhibition" conceived after the most approved fashion. It was most strange, in the midst of Stambul, to stumble upon this extraordinary anomaly, and to witness the turbaned Turk lounging curiously amidst the well-filled cases of Oriental wares, while a military band drowned the pleasant splash of a flower-imbedded fountain, in crashing polkas, waltzes, and quadrilles, to the infinite delectation of occasional specimens of ragged Moslemdom outside.

By the courtesy of the general commanding at Scutari, I was permitted to ramble at will through the barracks and hospital. I had stated my wishes to the officer on guard at the principal entrance of the barracks. He at once conducted me to the general's quarters, a room opening on the lower corridor, to the right. This room must have been used for stores during the British occupation of the building; for several roughly written labels, indicating the position of sundry articles of crockery, were still affixed to the walls. It was somewhat difficult, I am ashamed to say, to keep one's countenance in presence of the grave and dignified commandant. I had barely seated myself on the divan to which he had waved me, when my eye was caught by an inscription above his head. The walls of the apartment were scrupulously clean, and doubtless the inscription had been left, with its companion affixes, from that reverence which the Mussulman holds for written characters. But it referred to an article which, however great its utility in civilized life and its need in a large hospital, is commonly hid in the utmost privacy of a dwelling, and may not be mentioned to ears polite. The ludicrousness of the association, heightened by the profound humility of the satellites who thronged the room, was almost irresistible.

The labels in the general's quarters were the sole manifest reliques of the war which struck the eye in walking through the vast corridors and many of the rooms. The whole building was clean, and, as a rule, in good order. The rooms (it is difficult to avoid writing wards) I chiefly visited were occupied by a regiment which had recently returned from Montenegro, and which still retained the dress common to the Turkish soldiery in the time of the Crimean war. As many men were crowded into a room as the slightly raised floor upon which they slept would readily contain. Their carpets almost touched each other, after the old fashion so familiar to those who served with the Turks in the Russian war. True to their traditions also, the men had closed every accessible aperture of communication between the inner and the outer air. Excepting, perhaps, the ventilating openings which had been made between the rooms and the corridors, and the provisions for the perflation of the building by means of the staircases, all the efforts of the English medical officers to open the weary series of chambers to the fresh air had been summarily rendered of no effect.

There were some three thousand men in the building, but the corridors and the immense quadrangle were almost deserted, the men herding together in the rooms to escape a bitter wind laden with rain.

I wished to visit the portion of the barracks which had been occupied by Miss Nightingale, but it was closed up on account of some repairs going on there. A captain of infantry, who had been directed by the general commanding to accompany me, had served at Scutari, at the time the barracks were used as a hospital. I sought to learn from him, as well as from two or three old attendants in the general hospital, if any remembrance of a name and presence which, with the Englishman, constitute the most precious memory of the place, lingered with our former Moslem allies on the spot. They recalled vaguely the fact of the women nurses; but of the chiefest of these, not the utmost efforts of that best of dragomen in the "City of the Sultan," "Far-away Moses," could recall a remembrance. The puzzled countenances of the questioned was the clearest testimony to the sincerity of their replies, and afforded no inapt index of the bewilderment of these sons of Islam that a Giaour should so vex himself with womankind.

But the worthy captain became eloquent, as, leaving the interior of the barracks, we approached the cooking-kitchen, on the west side of the quadrangle, from the rear of which thin white columns of steam were straggling. He told me how every true believer who carried arms and solaced himself with the savoury messes which came from under the roof beneath us, mingled such blessings as a Mohammedan might mingle, with the pleasant odours that ascended heavenwards before his nostrils, for England's gift to the Turk of the splendid cooking-apparatus she had erected for her own sick and wounded in the great war. Hastening down the narrow steps from the quadrangle, and passing beneath the doorway of the kitchen, we plunged into a dense atmosphere of steam, and stumbled over a heap of rejected vegetable matter, damming up a wide lake of slush. The floor was swimming with waste water, and the walls were black and streaming with condensed steam. But the long range of splendid boilers in their white marble setting were in admirable order, with one exception, and all in use, each containing its bubbling burden of a grateful stew. The steam escape-pipe of one of the coppers had been detached, and hence the clouded atmosphere.

There were many tokens of neglect in the cooking-kitchen, the exterior of the barracks, and the drainage; and it seemed as if what my companion stated was correct, namely, that no systematic repairs had been carried out in the buildings since they had been vacated by the British. But he added, that in a little time all would be well, as it was known that the Sultan intended to place the whole of the structures, from foundation to roof, in as perfect order as when the English left them. This gossip is consistent with the changes and improvements which the Sultan has already made in the hygiène of his troops.

Crossing the open space to the south of the barracks, and dropping down the precipitous bank into the hollow beyond, I stopped for a moment at the wash-house, which exhibited little change. Then walking forwards, I gained the northern gates of the general hospital, before which was spread out a miniature slough. I remembered the hospital as a great block of red building, if my memory had not been playing me false; but now its outer walls were whitened, and they stood out clear and cold against the dark sky.

It would be difficult to exaggerate the exquisite order and cleanliness of the corridors and wards. To use a common Northern hyperbole expressive of great cleanliness, "One might have eaten from the floors." There were only a few patients in the building, but these were crowded into a small number of wards. The tendency to overcrowding is one of the greatest faults of the Turk; and here, in the hospital, it was as conspicuous as in the barracks. But the patients were placed on neat iron bedsteads; the bedding was excellent and superlatively clean; and the general arrangements visible on a passing glance, excepting only the shortcoming mentioned, very praiseworthy.

Leaving the hospital, I approached the most sacred point of my pilgrimage. The burial-place of our soldiers is situated on the summit of a low cliff overhanging the Sea of Marmora. One portion occupies a narrow strip of ground between the edge of the cliff and the boundary-wall running along the sea-face of the hospital; another portion occupies a green slope to the south of the hospital. When last I had visited the spot, the ground was unenclosed, the wild dogs roamed at will over it, and the graves were marked by rough weed-covered hillocks, with here and there a roughly-made wooden cross or simpler piece of smooth plank, inscribed with names, some of which even then were deciphered with difficulty.

Knocking at the gates, an old Turk admitted me within the enclosure. Neither the trim cottage of the English guardian and its pretty flower-beds, nor the gravelled walks which first met the eye as the gates swung back, nor the great memorial, with its grand sculptures, held the attention for a moment. I had thoughts only for the well-remembered mounds, which had changed but in this—that the slight wooden memorials of the war-time had given place to tall crosses, deeply-cut headstones, and heavy sarcophagi of pure and glistening marble.

A bitter north wind was blowing; the sky was hidden by heavy masses of black drifting cloud, which from time to time let fall a veil of penetrating rain; a thick haze circumscribed the horizon; the minarets and domes of Stambul hardly struggled through the flying mist; and the ruck of palaces, dwelling-houses, barracks, and time-worn walls, across the Bosphorus, lacking their setting of bright verdure, and with their many colours subdued by the dun atmosphere to one unvarying hue, looked black, wretched, and ruinous. Even Kadukoi, nestling beneath the hills on the beach to the left, did not escape from the dull monotony of gloom which enshrouded sea and shore, city and sky alike.

Standing out white and clear on the edge of the cliff, the long line of tombstones seemed as if they were keeping watch and ward over the green mounds beneath them—nay, it seemed almost as if in death, as in life, the officers stood at the head of their men, drawn up in the silent order of the grave—while above all brooded the solemn, impassive countenances of Marochetti's great granitic angels. Standing amidst the coarse grass which matted the grave-mounds, chequered with the brilliant white disks and bright yellow eyes of the ox-eyed daisy, and laden with wet, how vividly the memory recalled that sad time when every corridor and room of the vast buildings which overlooked the ground were blocked up with misery! How clearly rose before the mind, as in a dream, that weary succession of sick-freighted ships streaming southwards across the Euxine; while in the distant background stood out the wintry heights and plateaus of the Crimea, flecked with their hideous sloughs of human wretchedness. But amidst all the death-laden memories rested the bright vision of FLORENCE NIGHTINGALE—like to that celestial pilot whom Dante saw, "visibly written Blessed in his looks," caring for his charge of immortal souls in purgatory.

It was not without acute emotion, in which grief and pride were commingled, that I read on the faces of the tombstones the ineradicable testimony of the noble devotion of my own profession in the disaster of 1854-55. Most conspicuous among the memorials, and, if I err not, most numerous, were those to the hospital staff and to medical men. How familiar were the names of many who rested here in the close companionship of death!

DAVID ANDERSON,
*4th November*, 1854.

C. HUME READE,

*28th November*, 1854.

ALEXANDER STRUTHERS,
*20th January*, 1855.

EDMUND SIDNEY WASON,
*8th February*, 1855.

FREDERICK A. MACARTHY,

*12th February*, 1855.

JOHN GRAHAM,
*16th February*, 1855.

HARVEY LUDLOW,
*April*, 1855.

R. SIMONS,
*28th April*, 1855.

JAMES WISHART,
*25th May*, 1855.

ED. JOHN COMPLIN,
*25th October*, 1855.

ALEXANDER MAC GRIGOR,
*16th November*, 1855.

TERENCE H. WALL,*
*16th December*, 1855.

* H.M.S. *Leopard.*

DR. BROWN.

ASSISTANT-SURGEON H. W. WOOD.

DR. MAYNE.

DR. KEITTLE.*

SURGEON MACAULAY.†

SURGEON ROXALL.†

ASSISTANT-SURGEON SIBBALD.†

ASSISTANT-SURGEON COATES.†

DR. PARDOE.

Truly, a heavy sheaf has been gathered from our ranks, and garnered on this distant cliff by Death the Reaper.

There also I read the names of DISPENSER J. BEVERIDGE; SOPHIA BARNES, Nurse, April, 1855; SOPHIA WALFORD, Matron, 30th August, 1855; FANNY A. M. BIRD, 2nd September, 1855; MARTHA CLOUGH, 24th September, 1855 (died on the voyage from the Crimea to Scutari); MARY MARKS, 8th October, 1855; and CHARLOTTE MOORE, 22nd November, 1855.

It was impossible to gaze on these sad yet proud memorials, and not recall to memory that four of my own immediate companions during the war lay in their graves far away on the shores of the vexed Euxine.

RAY SAMUEL MILLARD,
*Died of Fever at Eupatoria, 9th June*, 1855.

LAWRENCE ORMEROD,
*Died of Cholera at Balaklava, 19th June*, 1855.

DISPENSER WILLIAM WALKINSHAW,
*Died of Cholera at Eupatoria, 19th July*, 1855.

WILLIAM FREDERICK EDSALL,
*Died of Fever at Trebizond, 13th January*, 1856.

Ormerod was buried in the vineyard which occupied the centre of the plateau on the heights east of Balaklava. All traces of his grave will doubtless, long ago, have been swept away by the wind and the rain. Standing on the spot where his remains lie, and looking towards the rising sun, the eye commands a scene consecrated in British and French history by blood. In the plain below the Russian cavalry charging upon the Highlanders—"*that thin red streak tipped with a line of steel*,"—had sped to destruction and the English heavy

* German Legion. † Anglo-Turkish Contingent.

cavalry had riven their enemies like a thunderbolt, on the 25th of October. The redoubt-capped ridge, running from left to right, hides the ground over which the light cavalry brigade rushed to its immortal defeat. Beyond rise the Fidukhine hills, overlooking the Tchernaya, burrowed with Russian "dug-outs," and cut in two by the gorge leading to Tractir bridge, the chief scene of the great fight of the 16th of August, 1855. A little to the right of these hills, across the river, springs the first spur of the highlands, thickly covered with brushwood, which stretch away to the east, until they join the heights of Ai-todor. On this spur was situated the Sardinian outpost, first attacked by the Russians at the battle of the Tchernaya. On the morning of that eventful day, I was sound asleep in my tent, on the hill-side above poor Ormerod's grave, when the noise of firing in an unaccustomed direction awoke me. Jumping off my bed, I threw back the flap of the tent. Early dawn was beginning to creep along the western slopes of the distant heights, and, low down, projected against the dark shadows which still enshrouded their roots in the plain, rose three tall, spectral columns of white smoke from the guns, the reports of which had awakened me. A moment more, and the rattle of musketry fell on the ear. Before noon the banks of the Tchernaya were cumbered with 3000 dead men, and a multitude of wounded. Beyond the Fidukhine hills the grass-grown plain of the Tchernaya spreads out until it reaches the base of the distant heights of Mackenzie, and the long line of tall white cliffs which close the horizon to the east, flashing back the rays of the sun in splendid radiance as it sinks to rest, and which at nightfall glittered with a bright crest of Russian watch-fires. The precipitous scarps of the wooded heights above Kamara rise close at hand on the right, and in the far left is seen the rugged cliff which forms the southern edge of the great plateau above Sebastopol, and the opening of the gorge of Inkermann, through which swelled the sound of the cannonade before the devoted city, multiplied by unnumbered echoes. In the spring of 1855 the plain of Balaklava was a gorgeous expanse of verdure chequered with wild flowers; in the summer it was an arid waste, torn up by the hoofs of horses and the wheels of ammunition-waggons and cannon, while its streams had been converted into deep sloughs by the wallowing of buffaloes. When the first advance on the Tchernaya took place on the 25th of May, riding across the portion of the plain over which the light cavalry charge had taken place, the tall, rich grass reaching to the saddle-girths, I came upon the skeleton of a horse and part of that of a man lying close together. The bones were bleached to a complete whiteness, but that which riveted the attention chiefly was that the cavity of the ribs had become a perfect nest of wild flowers, and a very mob of "quaint enamell'd eyes"—bright blue, red, yellow, and purple—peeped out of the interstices between the bones. It may be that Ormerod's resting-place is crowned each returning spring and summer with a crowd of blue and golden flowers; and it is a pleasant conceit to imagine that, in the expansion of their tender buds, we may read the

> "Emblems of our own great resurrection,
> Emblems of the bright and better land."

A mural monument in the church of the Lancashire village where Ormerod was born, perpetuates his memory and the regret of his comrades for his loss.

Millard and Walkinshaw were buried in the Jews' cemetery at Eupatoria; that famous cemetery, which early in the Crimean war was the scene of so fierce a struggle and so terrible a slaughter. It would be difficult to imagine a place more desolate. Behind it, the lines of earthworks that surrounded the town; to the right, the Great Salt Lake and the neighbouring sea; before, the bleak and trackless plain, stretching far away towards the kindred Steppes of Southern Russia, and without a house, or shrub, or tree, to vary its terrible monotony. Here and there, upon the horizon, might be seen huge mounds raised by some forgotten race. Much of the surface of the cemetery was ploughed up by shot, the monuments were broken and overturned, and the shallow graves of the country had, in many cases, yielded a scanty prey to vultures and starving curs. And beyond these monuments, when my colleagues were buried there, were hillocks marking new-made graves, in almost countless numbers. The proprietors of the cemetery, the Karaite Jews of Eupatoria, who before the invasion, were the most wealthy and prosperous of the inhabitants, were dying of starvation, and of the fever that starvation brings. As many as eighty deaths occurred in their community in a single day; and at all hours the miserable survivors might be seen tottering along towards the cemetery, sometimes hardly able to dig a grave little more than a foot deep, or to convey the corpses of their kindred to their last resting-places. Beside the remains of these innocent victims of the war, rest the bones of the two Englishmen. Their graves are guarded by carefully fenced monuments bearing inscriptions cut on marble by Tartar masons, who worked at the Roman characters to them unknown.

Two facts in connexion with Millard's death deserve to be recorded. The first is, that the Russian watcher of the dead at Eupatoria refused to watch by the corpse, alleging that the English were infidels, heathens, unfit to receive Christian observances at the hands of a son of the orthodox Eastern Church! The second that, when the funeral procession passed out of the gates on its way to the cemetery, the Turkish officer commanding the guard inquired who was dead. An English doctor, was the reply. "Oh!" said the worthy Mussulman; "is that all? There are plenty of them."

Edsall was buried in the cemetery of the Protestant Armenians at Trebizond. I have never seen the place where his remains lie. He was the last vicitm of the small staff of British surgeons serving with Omar Pasha's forces.

Ormerod, Millard, and Edsall were cut off in the very prime of life, and each had given rich promise of a bright career, but—

> "Their leaf has perish'd in the green
> And, while we breathe beneath the sun,
> The world, which credits what is done,
> Is cold to all that might have been."

Our dead rest in peace at Scutari and in the Crimea, but in their death an impulse arose, which, gaining rather than losing strength with the lapse of time, still works its way among us. The long and laborious inquiry of the Royal Commission on the Sanitary State of the Indian Army, which has just terminated, shows this—an inquiry which numbers among its co-operators the guiding spirit of the impulse at its commencement, FLORENCE NIGHTINGALE.

---

